# Clinical, Pathological, Laboratory Characteristics, and Treatment Regimens of Kimura Disease and Their Relationships With Tumor Size and Recurrence

**DOI:** 10.3389/fmed.2021.720144

**Published:** 2021-09-29

**Authors:** Lina Fan, Shiyan Mo, Yanyan Wang, Jian Zhu

**Affiliations:** ^1^Department of Rheumatology, Hainan Hospital of PLA General Hospital, Hainan, China; ^2^Department of Rheumatology, The First Medical Center of PLA General Hospital, Beijing, China

**Keywords:** eosinophilic lymphoid granuloma, Kimura disease, tumor recurrence, tumor size, treatment regimens

## Abstract

**Objective:** As of date, Kimura disease (KD) has an unclear etiology, no accepted diagnostic standard, and no definite treatment regimen. In this study, clinical and pathological laboratory characteristics and treatment regimens of patients with KD with different tumor sizes and status of tumor recurrence were analyzed. This was performed to identify the factors, which determine tumor size and recurrence, and to identify effective treatment methods for patients with KD.

**Methods:** A total of 33 hospitalized patients with a definite diagnosis of KD were enrolled in this study.

**Results:** There were 15 patients (45.5%) with a maximum tumor diameter of <3 cm. There were no statistically significant differences in age, gender, clinical symptoms, lesion sites, laboratory indicators, and treatment regimens among patients with a maximum tumor diameter <3 cm or ≥3 cm (*P* > 0.05). Among the 25 patients who completed the follow-up, there were 18 patients (72%) who had a recurrence of KD. There were no statistically significant differences in age, gender, clinical symptoms, the maximum tumor diameter, lesion sites, laboratory indicators, and initial treatment regimens between patients with or without the recurrence of KD (*P* > 0.05). There was a statistically significant difference in systolic blood pressure (SBP) between patients with or without the recurrence of KD (*P* < 0.05). All patients who received only surgical treatment had disease recurrence, 33.3% of patients who received prednisone therapy had no disease recurrence, and 37.5% of patients who received combination therapy showed recurrence.

**Conclusion:** The current study summarized clinical manifestations, pathological features, laboratory indicators, and treatment regimens of patients with KD. There were no significant differences in these aspects among patients with different tumor sizes, and there was no significant difference in these aspects except in the SBP between patients with or without the recurrence of KD, indicating that SBP is a significant clinical factor affecting disease recurrence in patients. Combination therapy with prednisone was found to be superior to surgical treatment.

## Introduction

Kimura disease (KD) is a benign, chronic inflammatory granuloma, also known as an eosinophilic lymphoid granuloma. It manifests mainly through progressive enlargement of a painless mass and is accompanied by the increased levels of eosinophils and immunoglobulin E (IgE) (1). As it is a rare disease in clinical practice, KD has an unknown etiology and unclear pathogenesis. It is difficult to distinguish KD from other types of inflammation, hemangiomas, and tumors, causing high rates of misdiagnosis. The treatment of KD is poor, and its recurrence rate is relatively high, which affects the quality of life and health of patients. A retrospective analysis of 25 patients with KD found that 60% of patients who received surgical treatment and 50% of patients who received drug treatment showed disease recurrence (2). The clinical manifestations, pathological features, laboratory characteristics, and treatment regimens of patients with KD related to tumor size and recurrence have been rarely reported in the previous studies. In the current study, clinical, pathological, and laboratory characteristics and treatment regimens of patients with KD with different tumor sizes and status of tumor recurrence were analyzed to identify factors influencing tumor size and recurrence and to identify reasonable and effective treatment methods in patients with KD.

## Materials and Methods

### Study Subjects

A total of 33 patients hospitalized with a definite diagnosis of KD were admitted to the Chinese People's Liberation Army (PLA) General Hospital from December 2003 to May 2019 (2). KD is clinically diagnosed as a benign and chronic inflammatory granuloma that manifests through progressive enlargement of a painless mass, eosinophil infiltration, and IgE elevation (1). The inclusion criteria were as follows: (1) definite KD diagnosis and local mass and (2) available clinical data and treatment regimens. Patients were excluded based on the following criteria: (1) those who could not cooperate with treatment regimens and (2) those who could not make timely visits to the hospital. The current study was approved by the Ethics Committee of the Chinese PLA General Hospital (Beijing, China). Written consent was obtained from each participant.

### Data Collection

Clinical characteristics, such as age, gender, and clinical symptoms, were collected based on standard procedures through in-person interviews by a well-trained research team from the Chinese PLA General Hospital. Weight was measured using a digital scale, and height was measured using a wall-mounted measuring tape, with patients wearing light clothes and no shoes. Body mass index (BMI) was calculated as weight (kg) divided by height squared (m^2^). Blood pressure was measured on the right arms of the participants with a calibrated desktop sphygmomanometer (Yuwell Medical Equipment and Supply Co., Ltd., Jiangsu, China). Appropriate cuff sizes were determined based on arm circumference. Participants sat in a chair for 5 min with their feet on the floor and their right arm supported at heart level. Systolic blood pressure (SBP) and diastolic blood pressure (DBP) were recorded at the first and fifth Korotkoff sounds. Samples of venous blood were routinely drawn by venipuncture and delivered to the Department of Biochemistry, Chinese PLA General Hospital. Qualified technicians who performed laboratory analyses were blinded to the clinical data. Ultrasound examination was performed to determine the diameter of the local mass. Pathological biopsy, hematoxylin-eosin staining, and immunohistochemical method were performed to determine histopathological changes, molecular markers and give a clear diagnosis of the local mass. Patients were contacted at regular intervals through a phone call and brought to our hospital to complete the 1-year follow-up. Follow-up could not be performed for eight patients; consequently, they were excluded from the study. A total of 25 patients completed the follow-up and received corresponding management.

### Statistical Analyses

Continuous data were expressed as mean and SD and compared between the two groups using Student's *t*-test. Categorical data were expressed as percentages and compared between the two groups with a Chi-squared test. *P* < 0.05 was considered statistically significant. Statistic Package for Social Science version 22 software was used for the statistical analyses.

## Results

Among the 33 patients in the current study, there were 28 males (84.8%) and five females (15.2%) aged 8–74 years with an average age of 39 years. The main lesions were either a painless soft tissue mass or a lymph node enlargement. There were six cases (18.2%) that involved a single region and 27 cases (81.8%) that involved multiple regions. The maximum tumor diameter upon performing an ultrasound ranged from 1.5 to 10 cm, with an average of 4.04 cm. There were 15 patients (45.5%) with a maximum tumor diameter <3 cm and 18 cases (54.5%) with a maximum tumor diameter ≥3 cm. As shown in Table 1, there is no statistically significant difference in age, gender, BMI, SBP, DBP, heart rate, clinical symptoms, lesion sites, and laboratory indicators between patients with a maximum tumor diameter of <3 cm or ≥3 cm (*P* > 0.05).

**Table 1:** Clinical features, laboratory indicators, and initial treatment regimens in patients with Kimura disease and different diameters of tumors.

**Characteristics**	**All (*n* = 33)**	**Tumor diameter <3 cm (*n* = 15)**	**Tumor diameter ≥3 cm (*n* = 18)**	***P* value**
Age (year)	391 ± 7	441 ± 8	351 ± 6	0.147
Gender (%)				0.790
Males	28 (84.8)	13 (86.7)	15 (83.3)	
Females	5 (15.2)	2 (13.3)	3 (16.7)	
BMI (kg/m^2^)	24.753 ± 0.64	24.293 ± 0.18	25.134 ± 0.03	0.513
Blood pressure				
SBP (mmHg)	1261 ± 3	1301 ± 1	1231 ± 4	0.120
SDP (mmHg)	829 ±	851 ± 0	798 ±	0.069
Heart rate (bp/min)	789 ±	795 ±	771 ± 2	0.601
Tumor diameter (cm)	4.042 ± 0.50	2.050 ± 0.38	5.692 ± 0.29	<0.001
Lesion site (%)				0.427
Parotid gland	10 (30.3)	5 (33.3)	5 (27.8)	
Neck	9 (27.3)	3 (20.0)	6 (33.3)	
Submandibular space	7 (21.2)	3 (20.0)	4 (22.2)	
Face	4 (12.1)	3 (20.0)	1 (5.6)	
Limb	2 (6.0)	0 [0]	2 (11.1)	
Lymphadenopathy	1 (3.0)	1 (6.7)	0 (0)	
Lesion number (%)				0.805
Single	6 (18.2)	3 (20.0)	3 (16.7)	
Multiple	27 (81.8)	12 (80.0)	15 (83.3)	
Symptom (%)				0.095
Skin itch	9 (27.3%)	2 (13.3)	7 (38.9)	
Fever	4 (12.1%)	1 (6.7)	3 (16.7)	
Thirst	3 (15%)	1 (6.7)	2 (11.1)	
Hoarseness	1 (6.7)	1 (6.7)	0 (0)	
Blurred vision	2 (6%)	1 (6.7)	1 (5.6)	
Laboratory indicators				
Leukocyte (10^9^/L)	8.252 ± 0.44	7.362 ± 0.05	8.992 ± 0.54	0.055
Eosinophil (10^9^/L)	0.210 ± 0.19	0.140 ± 0.09	0.260 ± 0.23	0.061
Elective CRP (%)	2 (6.1%)	0 (0)	2 (11.1)	0.183
Elective ESR (%)	1 (3%)	1 (6.7)	0 (0)	0.266
Initial treatment regimens				0.417
Single surgery	18 (54.5%)	8 (53.3%)	10 (55.5%)	
Single prednisone	7 (21.2%)	2 (13.3%)	5 (27.8%)	
Combined therapy	8 (24.3%)	5 (33.3%)	3 (16.7%)	

*BMI, body mass index; CRP, C-reactive protein; ESR, erythrocyte sedimentation rate; SBP, systolic blood pressure; SDP, systolic double products*.

**Figure 1:**
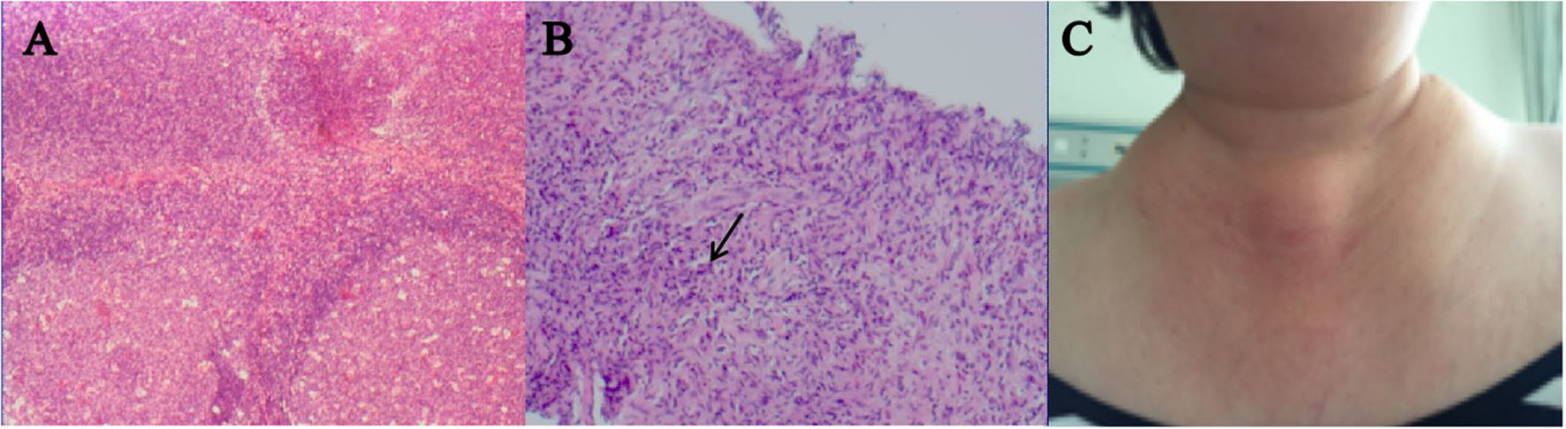
A 55-year-old woman with clinical features and pathological manifestations showing extensive lymphoid follicle-like structures and a large infiltration of eosinophilic granulocytes **(A,B)**, and local mass in the neck **(C)**. After receiving prednisone + cyclophosphamide, her tumor became smaller but as prednisone gradually decreased, the size of her tumor slightly increased. After receiving local radiotherapy again, this patient was suffering from pulmonary infection 3 months later and finally died due to multiple organ failure.

All patients had characteristic pathological manifestations, such as inflammatory cell proliferation and infiltration, extensive lymphoid follicle-like structures, a large number of eosinophilic granulocyte infiltration, formation of eosinophilic microabscesses, the proliferation of postcapillary venules, and varying degrees of fibrosis (Figures 1A,B) (3). Immunohistochemical results showed that the IgE positive rate was 27.3% (nine patients), IgG4 positive rate was 15.2% (five patients), Kappa positive rate was 6.1% (two patients), and Lambda positive rate was 3.0% (one patient). Other immunohistochemical markers are shown in Table 2. There were 18 patients (54.5%) who received only surgical treatment and seven patients (21.2%) who received only prednisone therapy. There were eight patients (24.3%) who received combined treatment, i.e., one patient (3.0%) who received surgery + prednisone, one patient (3.0%) who received surgery + prednisone + radiotherapy (15–20 gy), one patient (3.0%) who received surgery + radiotherapy (15–20 gy), one patient (3.0%) who received surgery + methotrexate + prednisone, one patient (3.0%) who received prednisone + cyclophosphamide + gamma globulin (20 mg, 3 d), and 3 patients (9.1%) who received prednisone + cyclophosphamide. There was no statistically significant difference in treatment regimens between patients with a maximum diameter of tumor <3 cm or ≥3 cm (*P* > 0.05).

**Table 2:** Positive rate of immunohistochemical molecules in Kimura disease.

**Molecules**	**IgE**	**IgG4**	**Kappa**	**Lambda**	**Bcl-6**	**Bcl-2**	**Ki-67**	**CD1a**	**CD3**	**CD10**
N (%)	9 (27.3%)	5 (15.2%)	2 (6.1%)	1 (3.0%)	4 (12.1%)	5 (15.2%)	15 (45.5%)	2 (6.1%)	15 (45.5)	5 (15.2)
Molecules	CD15	CD20	CD21	CD30	CD31	CD34	CD35	CD38	CD43	CD51
N (%)	13 (39.4)	12 (36.4)	7 (21.2)	3 (9.1)	2 (6.1)	5 (15.2)	1 (3.0)	2 (6.1)	4 (12.1)	11 (33.3)
Molecules	CD68	CD79	CD99	CD117	CD138	CK18	EMA	GCDFP-15	CK5	CK
N (%)	5 (15.2)	2 (6.1)	1 (3.0)	1 (3.0)	2 (6.1)	1 (3.0)	1 (3.0)	1 (3.0)	1 (3.0)	1 (3.0)
Molecules	P63	S-100	SMA	CEA	PSA	PTAH	PAX-5	AS	ALK	
N (%)	1 (3.0)	7 (21.2)	1 (3.0)	3 (9.1)	1 (3.0)	1 (3.0)	2 (6.1)	2 (6.1)	2 (6.1)	

*Ig, immunoglobulin; Bcl, B cell lymphoma; CD, cluster of differentiation; EMA, epithelial membrane antigen; GCDFP-15, gross cystic disease fluid protein 15; CK5, cytokeratin 5; SMA, smooth muscle antibody; CEA, carcinoma embryonic antigen; PSA, prostate-specific antigen; PAX-5, B cell-specific transcription factor; AS, acid-fast staining; ALK, anaplastic lymphoma kinase*.

*Pathological biopsy and immunohistochemical methods were applied to determine the positive rate of the molecular mark of local mass*.

Among the 25 patients who completed the follow-up, there were 18 patients (72%) with a recurrence of KD. As shown in Table 3, there are no statistically significant differences in age, gender, BMI, DBP, heart rate, clinical symptoms, the maximum diameter of tumor, lesion sites, laboratory indicators, and initial treatment regimens between patients with or without recurrence of KD (*P* > 0.05). There was a statistically significant difference in SBP between patients with or without recurrence of KD (*P* < 0.05). All patients who received only surgical treatment had disease recurrence, 33.3% of patients who received prednisone therapy had no disease recurrence, and 37.5% of patients who received combination therapy showed recurrence. There was no statistically significant difference in the initial treatment regimen between patients with or without tumor recurrence (*P* > 0.05). The tumor of one patient became smaller after a long-term treatment with prednisone + cyclophosphamide. However, the tumor slightly increased in size after prednisone levels eventually decreased. After receiving local radiotherapy again, this patient was suffering from a severe pulmonary infection 3 months later and finally died owing to multiple organ failure (Figure 1C). Another patient underwent pathological biopsy and immunohistochemical analysis after the recurrence of the tumor so as not to exclude the possibility of transformed or combined lymphoma.

**Table 3:** Characteristics of patients with Kimura disease with or without recurrence.

**Characteristics**	**With recurrence (*n* = 18)**	**Without recurrence (*n* = 7)**	***P* value**
Age (year)	351 ± 8	461 ± 1	0.149
Gender (%)			0.884
Males	15(83.3)	6(85.7)	
Females	3(16.7)	1(14.3)	
BMI (kg/m^2^)	23.703 ± 0.57	25.372 ± 0.93	0.285
Blood pressure			
SBP (mmHg)	1201 ± 1	1311 ± 3	0.041
SDP (mmHg)	801 ± 0	841 ± 1	0.375
Heart rate (bp/min)	771 ± 1	786 ±	0.860
Tumor diameter (cm)	4.882 ± 0.44	3.293 ± 0.00	0.181
Lesion site (%)			0.727
Parotid gland	5 (27.8)	2 (280.6)	
Neck	7 (38.9)	1 (14.3)	
Submandibular space	2 (11.1)	2 (28.6)	
Face	2 (11.1)	1 (14.3)	
Limb	1 (5.6)	1 (14.3)	
Lymphaden	1 (5.6)	0 (0)	
Lesion number (%)			0.656
Single	4 (22.2)	1 (14.3)	
Multiple	14 (77.8)	6 (85.7)	
Symptom (%)			0.186
Skin itch	5 (27.8)	2 (28.6)	
Fever	3 (16.7)	0 (0)	
Thirst	3 (16.7)	0 (0)	
Hoarseness	0 (0)	1 (14.3)	
Blurred vision	0 (0)	1 (14.3)	
Laboratory indicators			
Leukocyte (10^9^/L)	7.802 ± 0.23	8.802 ± ,31	0.330
Eosinophil (10^9^/L)	0.180 ± 0.20	0.260 ± 0.14	0.360
Elective CRP (%)	2 (11.0)	0 (0)	0.358
Elective ESR (%)	1 (5.6)	0 (0)	0.524
Initial treatment regimens (%)			0.213
Single surgery	6 (33.3)	0 (0)	
Single prednisone	2 (11.1)	1 (14.3)	
Combined therapy	10 (55.6)	6 (85.7)	
Treatment regimens after recurrence (%)			<0.001
Single surgery	9 (50.0%)	0 (0)	
Single prednisone	2 (11.1%)	0 (0)	
Combined therapy	7 (38.9%)	0 (0)	
No therapy	0 (0)	7 (100)	

*CRP, C-reactive protein; ESR, erythrocyte sedimentation rate; SBP, systolic blood pressure; SDP, systolic double products*.

## Discussion

Kimura disease was reported by Kimura from southern Japan, with unclear etiology and pathogenesis (4). KD may be related to injured body tissue, disordered immune system, virus infection, tuberculosis (TB) infection, and hypersensitivity reactions (5–7). In the current study, four patients had a history of pulmonary infection, TB, and hepatitis B before the diagnosis of KD. The diseases in these cases were not found to be in the active stage. However, considering the chronic carrying course of these diseases, it cannot be concluded that the onset of KD was not due to the low-toxicity state of these diseases. Owing to the limited data in this study and the lack of statistical significance from big data, it is necessary to further explore whether infectious factors have a certain significance in the etiology and pathogenesis of KD.

There is no internationally recognized standard for the diagnosis of KD. Based on the existing studies, a comprehensive diagnosis of KD should be made according to clinical features, laboratory examination, and pathological features. KD is characterized by eosinophilic granulocyte infiltration and elevated IgE levels. There was an IgE positive rate of 27.3% in the current study. Thus, identifying eosinophilic lymphoid granuloma after the histopathological examination is crucial for diagnosing KD. In the current study, all patients with KD had characteristic pathological manifestations, such as inflammatory cell proliferation and infiltration, extensive lymphoid follicle-like structures, a large infiltration of eosinophilic granulocytes, formation of eosinophilic microabscesses, proliferation of postcapillary venules, and various degrees of fibrosis. Besides, it should be noted that there was an IgG4 positive rate of 15.2% in the current study. However, it is not clear whether KD is related to IgG4-related diseases (IgG4-RD). Previous studies have found clinical and pathological similarities between patients with KD and 27 published cases of IgG4-RD (8, 9). Pathological manifestations of IgG4-RD are plasmacyte infiltration, thrombophlebitis, or typical fibrosis, accompanied by elevated IgG4 levels. Owing to the limited data at present, it is very difficult to distinguish between KD and IgG4-RD, and their relationship needs to be explored in the future.

The recurrence rate of KD is about 60–80%, based on the previous literature (5). Our findings are consistent with the abovementioned recurrence rate, as the current study found that the recurrence rate was 72% (18/25) in the 25 patients for whom follow-up was completed. The current study identified that the SBP in the recurrent group was significantly different from that in the non-recurrent group, suggesting that SBP might be a key protective factor for the recurrence of KD. The higher the SBP is, the lower the risk of recurrence. The relationship between SBP and KD recurrence is worthy of further exploration through a multi-center study with more data. The pathomechanisms for the relationship between the higher SBP and the lower risk of recurrence are unclear and should therefore be clearly researched in further studies. The current study also found that there were no statistically significant differences in the maximum tumor diameter between the recurrent and non-recurrent groups, but the maximum tumor diameter in the recurrent group was still relatively larger than that in the non-recurrence group. This finding needs to be clarified by further studies with larger sample sizes. In the current study, one patient was suffering from a severe and unmanageable pulmonary infection and finally died due to a multiple organ failure. Pathological biopsy and immunohistochemical analysis did not rule out complications or transformation to lymphoma in patients after the recurrence of the tumor. One patient in this study showed complications, such as pulmonary infection and multiple organ failure, which reminds us that even if literature shows that KD has a relatively good prognosis (6–8), there is still a need to keep a watchful eye on the risk of transformation to malignant tumors and development of KD as a consequence of other diseases.

At present, there is no consensus on the treatment options for patients with KD. Surgical resection is often applied as the first choice for the treatment of single focal tumors. Other treatment methods are oral drug administration and radiotherapy (6–8). Oral prednisone and immunosuppressants might be effective for KD, and long-term maintenance therapy could prevent the recurrence of KD (10). Patients were prone to recurrence after the reduction or withdrawal of prednisone. In the current study, although there was no statistically significant difference in initial treatment regimens between patients with and without the recurrence of tumor, it suggested that prednisone and its combined therapy may be superior to surgical treatment alone in preventing the recurrence of KD. Some studies support the claim that a small dose of local radiotherapy provided a more effective treatment for KD (11). Simple surgical treatment has no obvious advantage compared with single radiotherapy for controlling local recurrence. A meta-analysis study found that only after surgical treatment, KD had a 5 times higher risk of recurrence compared to those undergoing radiotherapy after the operation (7). In other words, surgical treatment combined with postoperative radiotherapy has a lower risk of recurrence than surgery alone or radiotherapy alone. Studies have also found that patients with KD who received a single cycle of prednisone therapy as an initial treatment regimen had a high recurrence rate after the reduction or withdrawal of prednisone, and it was necessary to additionally administer radiotherapy after the recurrence of the tumor (12). It has been reported previously that combination therapy is superior to any individual treatment regimen (12). The current study found that prednisone remains the cornerstone of treatment for patients with KD, regardless of whether it is combined with other therapies. It is suggested that patients with KD should take prednisone as the basis of treatment and choose surgical treatment and postoperative combined immunosuppressor therapy to further control the progression and recurrence of KD. Based on the condition and tolerance of the patience, combined immunosuppressor therapy or local radiotherapy could be chosen after surgery.

The current study has both advantages and limitations. This study examined as many as 33 cases of KD, a very rare disease. Since diagnostic criteria and therapeutic methods are not fully understood in this disease, the current study may help to provide significant clinical data. However, the sample size of this study is limited, especially for the patients who are included in the follow-up.

## Conclusions

As of date, KD has an unclear etiology, no accepted diagnostic standards, and no definite treatment regimens. Treatment is mainly guided by the published literature and clinical experience. The current study summarized clinical manifestations, pathological features, laboratory indicators, and treatment regimens of patients with KD. We found that there were no significant differences in these aspects among patients with different tumor sizes and no significant difference in these aspects except in the SBP between patients with or without recurrence of KD, indicating that SBP is a significant clinical factor affecting disease recurrence. Combination therapy with prednisone was found to be superior to the surgical treatment. This study provides valuable insight for clinical doctors in the intervention of risk factors and selection of treatment regimens for KD, with the potential to promote better prevention, diagnosis, and treatment of disease recurrence.

## Data Availability Statement

The original contributions presented in the study are included in the article/supplementary material, further inquiries can be directed to the corresponding author/s.

## Ethics Statement

The studies involving human participants were reviewed and approved by Ethics Committee of PLA General Hospital. The patients/participants provided their written informed consent to participate in this study.

## Author Contributions

LF, SM, YW, and JZ designed, performed, and wrote this manuscript. All authors contributed to the article and approved the submitted version.

## Conflict of Interest

The authors declare that the research was conducted in the absence of any commercial or financial relationships that could be construed as a potential conflict of interest.

## Publisher's Note

All claims expressed in this article are solely those of the authors and do not necessarily represent those of their affiliated organizations, or those of the publisher, the editors and the reviewers. Any product that may be evaluated in this article, or claim that may be made by its manufacturer, is not guaranteed or endorsed by the publisher.
